# Smartphone-Based Monitoring of Parkinson Disease: Quasi-Experimental Study to Quantify Hand Tremor Severity and Medication Effectiveness

**DOI:** 10.2196/21543

**Published:** 2020-11-26

**Authors:** Elina Kuosmanen, Florian Wolling, Julio Vega, Valerii Kan, Yuuki Nishiyama, Simon Harper, Kristof Van Laerhoven, Simo Hosio, Denzil Ferreira

**Affiliations:** 1 University of Oulu Oulu Finland; 2 University of Siegen Siegen Germany; 3 University of Manchester Manchester United Kingdom; 4 University of Tokyo Tokyo Japan

**Keywords:** Parkinson disease, smartphone, hand tremor, mobile health

## Abstract

**Background:**

Hand tremor typically has a negative impact on a person’s ability to complete many common daily activities. Previous research has investigated how to quantify hand tremor with smartphones and wearable sensors, mainly under controlled data collection conditions. Solutions for daily real-life settings remain largely underexplored.

**Objective:**

Our objective was to monitor and assess hand tremor severity in patients with Parkinson disease (PD), and to better understand the effects of PD medications in a naturalistic environment.

**Methods:**

Using the Welch method, we generated periodograms of accelerometer data and computed signal features to compare patients with varying degrees of PD symptoms.

**Results:**

We introduced and empirically evaluated the tremor intensity parameter (TIP), an accelerometer-based metric to quantify hand tremor severity in PD using smartphones. There was a statistically significant correlation between the TIP and self-assessed Unified Parkinson Disease Rating Scale (UPDRS) II tremor scores (Kendall rank correlation test: z=30.521, *P*<.001, τ=0.5367379; n=11). An analysis of the “before” and “after” medication intake conditions identified a significant difference in accelerometer signal characteristics among participants with different levels of rigidity and bradykinesia (Wilcoxon rank sum test, *P*<.05).

**Conclusions:**

Our work demonstrates the potential use of smartphone inertial sensors as a systematic symptom severity assessment mechanism to monitor PD symptoms and to assess medication effectiveness remotely. Our smartphone-based monitoring app may also be relevant for other conditions where hand tremor is a prevalent symptom.

## Introduction

### Background

Parkinson disease (PD) is a neurodegenerative condition that affects patients’ physical and mental health [1,2] and has a wide variety of motor and nonmotor symptoms. Tremor is a cardinal motor symptom that can heavily hinder patients’ quality of life [3] and is generally defined as an involuntary, rhythmic, oscillatory movement of a body part [4]. Tremor can be categorized based on its activation conditions into rest and action tremor; in turn, action tremor is further divided into kinetic, postural, or isometric subtypes [4]. Rest tremor affects body parts that are not being voluntarily activated [4], kinetic tremor appears during any voluntary movement, postural tremor presents while maintaining a posture against gravity [5], and isometric tremor occurs during a muscle contraction against a rigid surface [6].

Among patients with PD, approximately 75% suffer from rest tremor, around 50% from moderately severe postural tremor [7], and an undetermined percentage from kinetic tremor [6]. These three types of tremor are pivotal in understanding PD. Typically, the amplitude of rest tremor decreases when patients activate the affected muscles and increases during mentally stressful situations [4,8]. We target hand rest, postural, and kinetic tremor, which occur at different frequency ranges (3-6 Hz, 6-9 Hz, and 9-12 Hz, respectively) [5]. The severity of PD tremor is usually assessed visually by a health professional during clinical visits, using tools such as the Unified Parkinson Disease Rating Scale (UPDRS) [9]. Recently, however, researchers have investigated the use of unobtrusive and objective sensing technologies to detect and quantify hand tremor.

Dyskinesia is defined as involuntary movement, different from tremor, and is related to the timing and dosage of levodopa medication [10]. We refer to a movement in the 1-3 Hz frequency range as dyskinesia [11].

### Related Work

Accelerometer data have been used to assess hand tremor using smartphones [11-17] and wearable devices [5,18-20]. Previous studies have attached an iPhone (Apple Inc) to a glove and collected data using the smartphone’s built-in accelerometer [11,12,15]. In a study by LeMoyne et al [11], subjects were asked to use the glove while extending their forearm for 10 seconds. The study found a statistically significant difference in the frequency response of the acceleration signal between a participant with PD and one without. Barrantes et al [12] measured rest and postural tremor in 30-second episodes and identified relevant accelerometer features to classify PD tremor and essential tremor. Similarly, Duque et al [15] collected accelerometer data with participants at rest and with their arms extended, and utilized machine learning to classify PD and essential tremor.

Bazgir et al [16] used a similar setup with a glove to classify rest, postural, and kinetic tremor UPDRS scores based on accelerometer data logged during three scripted 1-minute tests: at rest, with arms stretched, and while touching their nose with their index finger. Kostikis et al [14] used a glove-mounted smartphone to quantify rest and postural tremor severity. They found a statistically significant difference between healthy participants and participants with PD, but not between the left and right hands of people with PD. In addition, they studied the effects of PD medications in two volunteers with PD using accelerometer data collected in “on” and “off” medication states in laboratory conditions. The measurements were taken for the right and left hands separately, at rest, and with their hands extended. The “off” measurement was taken right before medication intake, and the “on” measurement was taken 1 hour after intake. The researchers detected a decrease in the metrics (the sums of the squared magnitudes of the acceleration and the sum of absolute differences in the acceleration vector) after medication intake, with the exception of the right-hand extended task for one of the volunteers.

Although these previous experiments have had positive results on quantifying tremor, we believe that the utility of the findings outside of the laboratory or a health care facility is limited. The practicality of carrying and wearing a glove at all times is up for debate, especially under extreme weather conditions. Accordingly, a smartphone-only solution was first investigated by Woods et al [13], where subjects held a smartphone for 10 seconds with their arm perpendicular to their body and elbow pointing out under six conditions: with eyes open, with eyes closed, during a bubble-balancing task, during a laser-pointing task with two different distances, and while counting backward by decrements of three. The authors detected a statistically significant difference in the accelerometer signal between a group with PD and a group with essential tremor, but they focused on the effects of the six cognitive tasks on tremor. Following up this line of research, this paper explores the difference in the accelerometer signal captured during a scripted task without additional hardware other than a smartphone.

In our study, the data were collected in naturalistic settings using a mobile toolkit, the Sentient Tracking of Parkinson’s (STOP) app, for monitoring PD symptoms in daily life. STOP includes a gamified tremor assessment module based on a ball-balancing game that logs the smartphone’s accelerometer, gyroscope, and rotation data. STOP also provides users with a medication intake journal and a daily symptom survey mechanism [21-23]. The data set used in this paper was published previously [21]. In this study, we analyzed the data set’s accelerometer and medication intake data to answer the following research questions: (1) how feasible is it to characterize hand tremor using inertial data captured during our smartphone game?, and (2) can the effects of PD medication be detected using the same inertial data captured during game sessions played before and after medication intake?

## Methods

We installed the STOP app into the smartphones of 13 participants diagnosed with PD and collected accelerometer data and medication logs. We used the Welch method to generate the power spectral densities (PSDs) and extracted features from the accelerometer data that we used to investigate the feasibility of hand tremor assessment and medication effect.

### STOP Application and Data Collection

STOP is a smartphone app developed for people with PD with four core functionalities: (1) an accelerometer-based ball game for quantifying patients’ hand tremor, (2) a medication journal for logging medication intake times, (3) a daily survey for reporting the overall severity of PD symptoms, and (4) reminder notifications [21-23].

To play the ball game, one has to place the smartphone horizontally on the palm of the hand for 10 seconds and try to keep a virtual ball inside a circle at the center of the screen. During the game session, STOP logs data from the accelerometer, linear accelerometer (acceleration without gravity’s influence), gyroscope, and rotation vector sensors [24]. It also records the position of the ball in relation to the inner circle’s center and the screen’s pixel density to compute an adjusted distance between the center of the ball and the center of the screen. The inertial sensors’ sampling frequency is set to 50 Hz (or the device’s maximum, if less than 50 Hz). In addition, users can record their medication intake using the “now” or “specify time” buttons, or with their voice via the natural language processing provided by Wit.ai [25].

During a real-world trial of STOP, data were collected from 13 participants with PD, eight females and five males [21]. The participants were recruited from two countries, seven from Finland and six from the United Kingdom. In this study, we had to exclude two participants because of poor data quality (see Data Set). Table 1 provides a summary of the remaining 11 participants’ characteristics; more details are provided in Multimedia Appendix 1. Participants were asked to install and use the STOP app for 1 month on their personal smartphones (five iPhones and six Android phones) and to participate in three interviews (at the start of the study, midway, and at the end).

Participants from Finland were located around the country, and their consent to participate in the study was given via the application. Participants from the United Kingdom, on the other hand, signed a paper consent form. Following local guidelines, approval from the University of Oulu’s ethical committee was not needed because the risks associated with participating in the study were similar to those of daily smartphone use. In previous publications, we have shared users’ experience during the trial and an analysis of the interview data [21]. To summarize, participants were willing to use digital tools to track their condition and were open to the possibility of sharing their data with their doctors, which functioned as a motivator to use such tools. In this paper, we analyze the inertial sensor data recorded during the game sessions and medication logs to quantify the severity of hand tremor and the effect that medication has on it.

**Table 1:** Overview of participants’ characteristics.

Characteristics		Participants (n=11)	
Age (years), mean (range)		64.7 (52-73)	
Years since PD diagnosed, mean (range)		7.1 (2-17)	
Number of PD medications, mean (range)		3 (1-5)	
Number of total daily medications^a^, mean (range)		4.3 (1-7)	
UPDRS II score^b^, mean (range)		11.8 (3-31)	
Tremor on UPDRS, mean (range)		1.2 (0-3)	
Deep brain stimulator installed, n (%)		2 (18%)	
Suffer from hand tremor, n (%)		4 (36%)	
Plays with tremor-affected hand, n (%)		2 (18%)	
Suffers from other issues affecting hands, n (%)			
	Rigidity		3 (27%)
	Bradykinesia		1 (9%)

^a^Refers to the number of medication intake times (ie, how many times per day the participant has to take medications, one or several at a time).

^b^The Unified Parkinson Disease Rating Scale (UPDRS) II score quantifies the severity level of Parkinson disease (PD) symptoms affecting daily activities (maximum score of 52). The scale for the tremor item on the UPDRS is as follows: 0=no tremor, 1=slight and infrequently present tremor, 2=moderate and bothersome tremor, 3=severe tremor interfering with many activities, and 4=marked tremor interfering with most activities.

### Data Set

Our data set contained a total of 1856 medication logs (mean of 107 [SD 54.9] logs per participant) and 2213 game sessions (mean of 138 [SD 60.6] sessions per participant). These data were recorded in 13 participants (P01 to P13) in naturalistic conditions. Participants had varying medication regimens.

Game sessions were 10 seconds long. We excluded P03’s sessions because the accelerometer sampling rate of his smartphone was approximately 25 Hz instead of the desired 50 Hz, and we excluded P04’s sessions because they only contained one sensor sample throughout the entire game for unknown technical reasons. P05’s phone had data synchronization issues, so only 1 week of data was collected, and P07 missed the first week of data collection because he had problems installing the application. Despite this, we included P05 and P07 in the analysis, resulting in a total of 11 participants.

Because the data were collected during a trial deployment of the STOP app, there were no participant exclusion criteria related to PD symptom severity. Based on the UPDRS II tremor self-reports, we categorized the participants into five groups: (1) all participants: P01, P02, P05, P06, P07, P08, P09, P10, P11, P12, and P13; (2) no tremor (participants reported no tremor): P02, P09, and P11; (3) tremor (participants reported tremor but in an unspecified location): P06, P07, P08, and P12; (4) hand tremor (participants reported hand tremor and played with the unaffected hand): P01 and P05; and (5) plays with hand tremor (participants reported hand tremor and played with the affected hand): P10 and P13.

We highlighted individual circumstances that might affect STOP’s measurements. P02 reported that his hand rigidity helped him to keep the ball still during game sessions. P09 had poor rotation in his wrists and P11 suffered from rigidity and bradykinesia that make him feel stiff and slow, which might have had a similar effect to that of P02. Finally, P01 was right-handed but used his left hand for playing. Multimedia Appendix 1 provides more details about participants’ symptoms and playing conditions.

### Data Preprocessing

**Figure 1:**
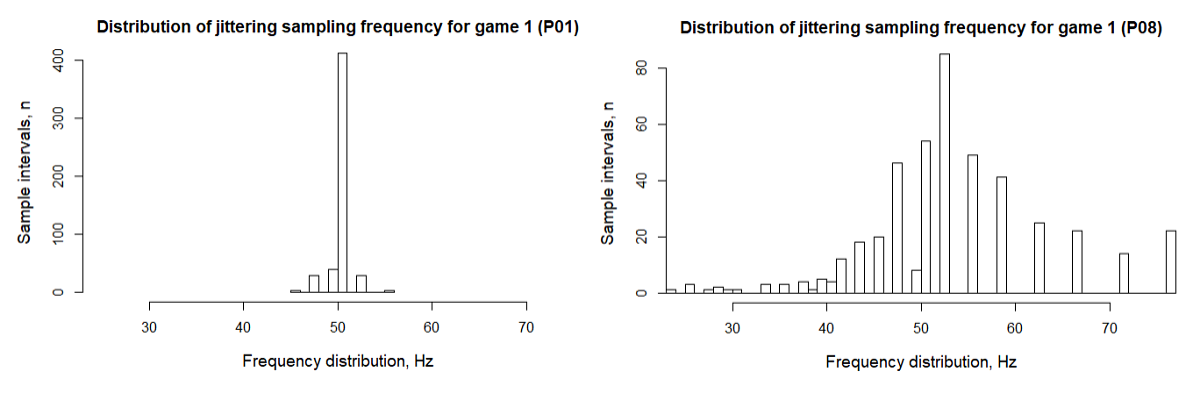
Best case (left) and worst case (right) examples of varying smartphone sampling frequency during a game session.

Accelerometer data were recorded as participants played a game for a duration of 10 seconds, henceforth referred to as a “game session.” The accelerometer sampling rate was set to 50 Hz, but the sampling rate varied across different smartphones, as the participants used their own devices for the study. In addition, for some devices, the sampling rate varied within a game session. In the examples in Figure 1, P01’s sampling rate stayed close to the requested rate, while the rate varied in P08’s device. For all devices, the sampling frequency was set to 50 Hz. To address the variation in the sensors’ sampling rate and get uniformly sampled data, we applied a linear interpolation on the accelerometer signal (see Multimedia Appendix 2 for technical details).

**Figure 2:**
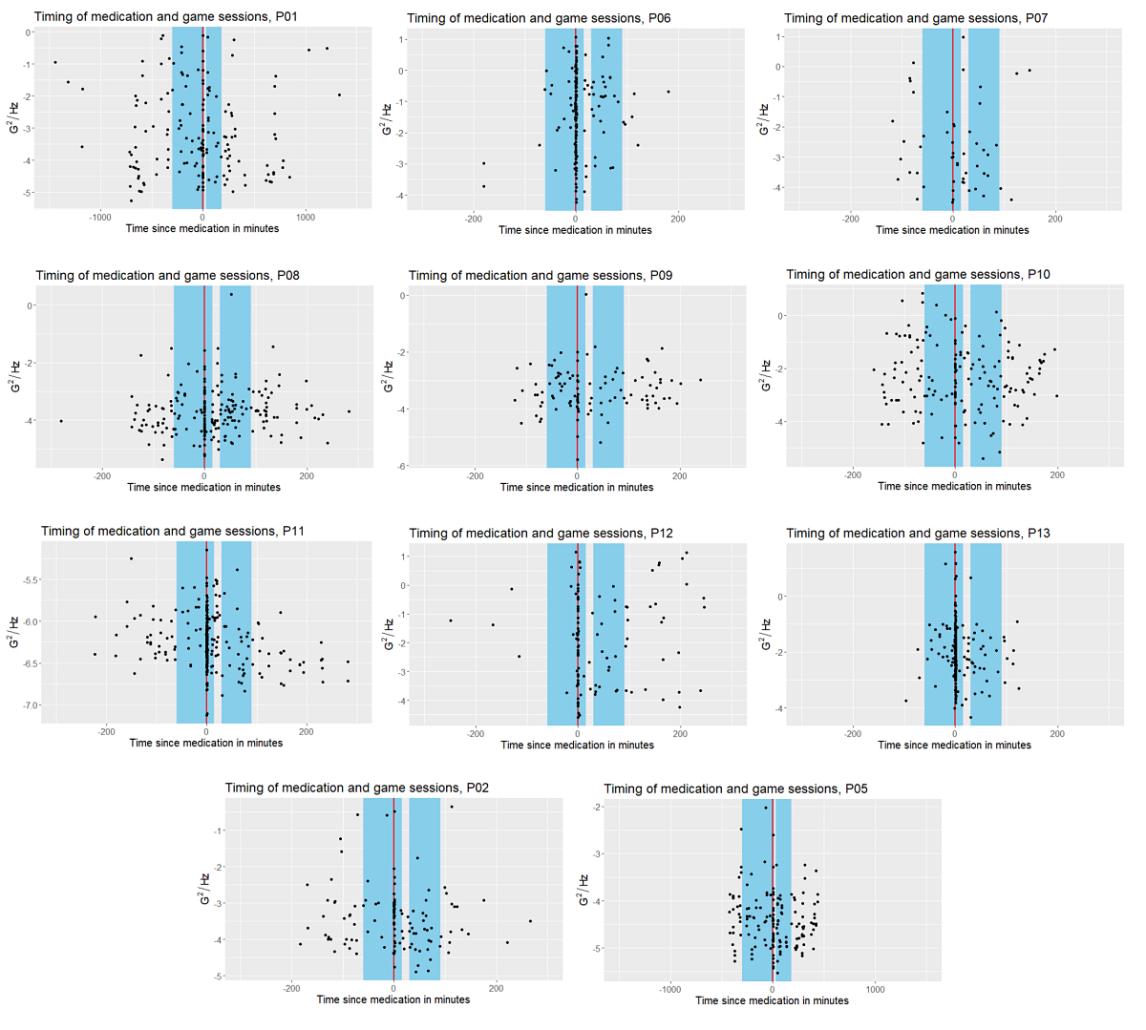
The timing of medication intake and game sessions. The x-axis shows the time since medication, 0 is the medication intake time and is highlighted with a red vertical line. Each game is associated with the closest medication intake time, either before or after. The y-axis presents the acceleration signal power in logarithmic scale; the sum of power is calculated over the entire spectrum for each game session. Note that the y-axis ranges differ. The first three rows show participants with more than two intakes per day while the last row shows those with only one or two.

We identified the closest medication intake record to each game session and labeled the game sessions as “before” or “after” (see Figure 2). Depending on multiple factors, medication can take at least 15 minutes to kick in [26]. Therefore, for participants who had to take their medication once or twice per day (every 24 or 12 hours), a game session was considered “before” medication if it was played between 5 hours before or 15 minutes after the intake log was completed. In contrast, the game session was labeled “after” medication if it was played between 30 minutes and 3 hours after the medication intake log was completed. For participants with more than two medication intakes per day, we used shorter thresholds. Specifically, the game session was labeled “before” if it was played either 1 hour before or 15 minutes after the medication intake, and “after” if it was played between 30 and 90 minutes after the medication intake. Game sessions outside of these periods were not included in our medication effect analysis. Figure 2 shows an overview of the labeled time periods; the medication intake time is denoted as a red solid vertical line centered around zero.

### Frequency Analysis: PSD

PD symptoms can be observed in specific frequency bands: dyskinesia (1-3 Hz), rest tremor (3-6 Hz), postural tremor (6-9 Hz), and kinetic tremor (9-12 Hz). As described in the introduction, tremor can be classified by its activation condition. In our study setup, depending on the user’s posture during a game session, we expected to see differences in the accelerometer signal in rest tremor, postural tremor, and dyskinesia frequencies, which we tried to detect by analyzing this signal in the frequency domain.

**Figure 3:**
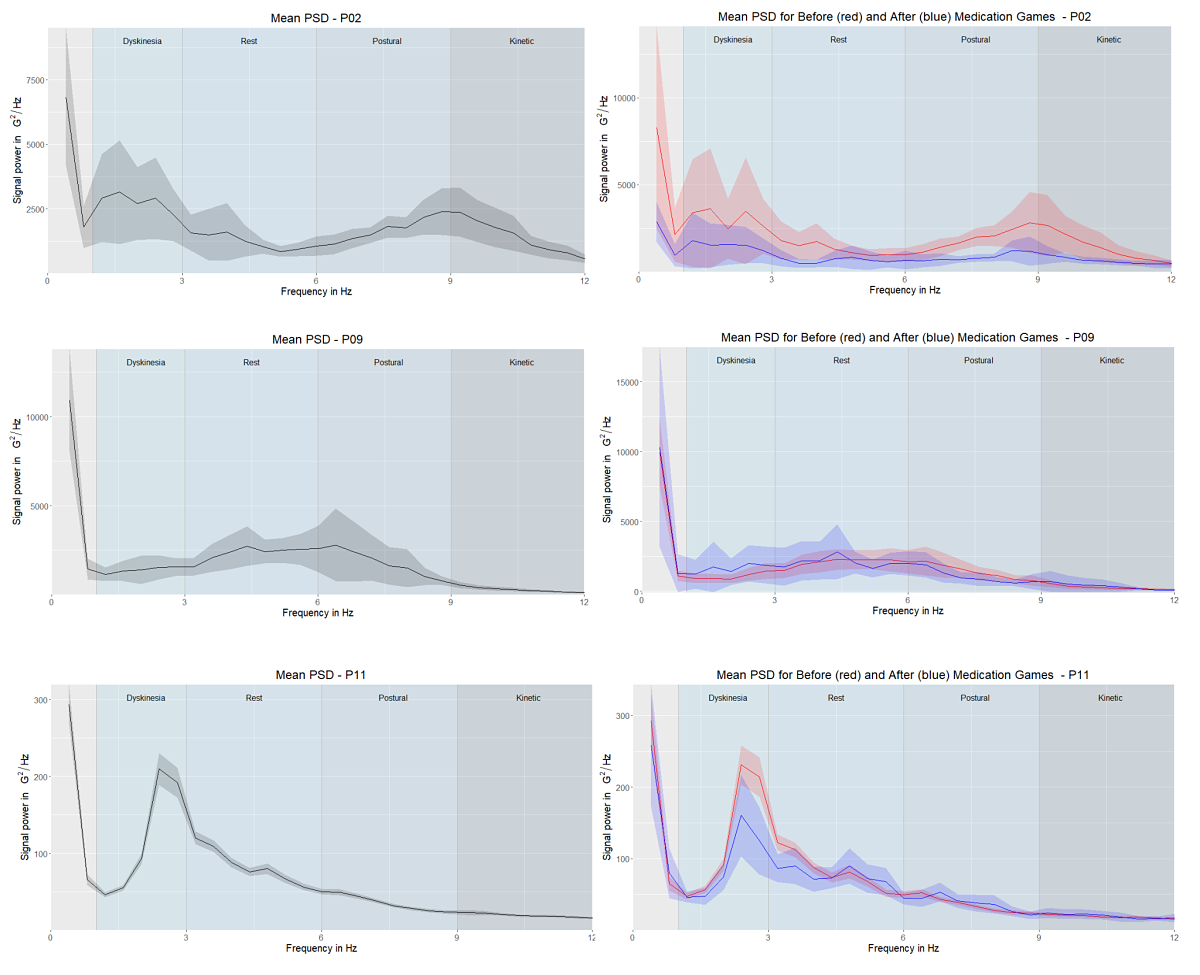
Mean of the power spectral densities with the 95% CI for the “no tremor” group (P02, P09, and P11). The left column shows the mean of all game sessions, and the right column shows the mean of the power spectral densities for “before” (red) and “after” (blue) games. Note that the y-axis ranges differ. Frequency areas (dyskinesia, rest tremor, postural tremor, and kinetic tremor) are denoted by different column shades of the background.

**Figure 4:**
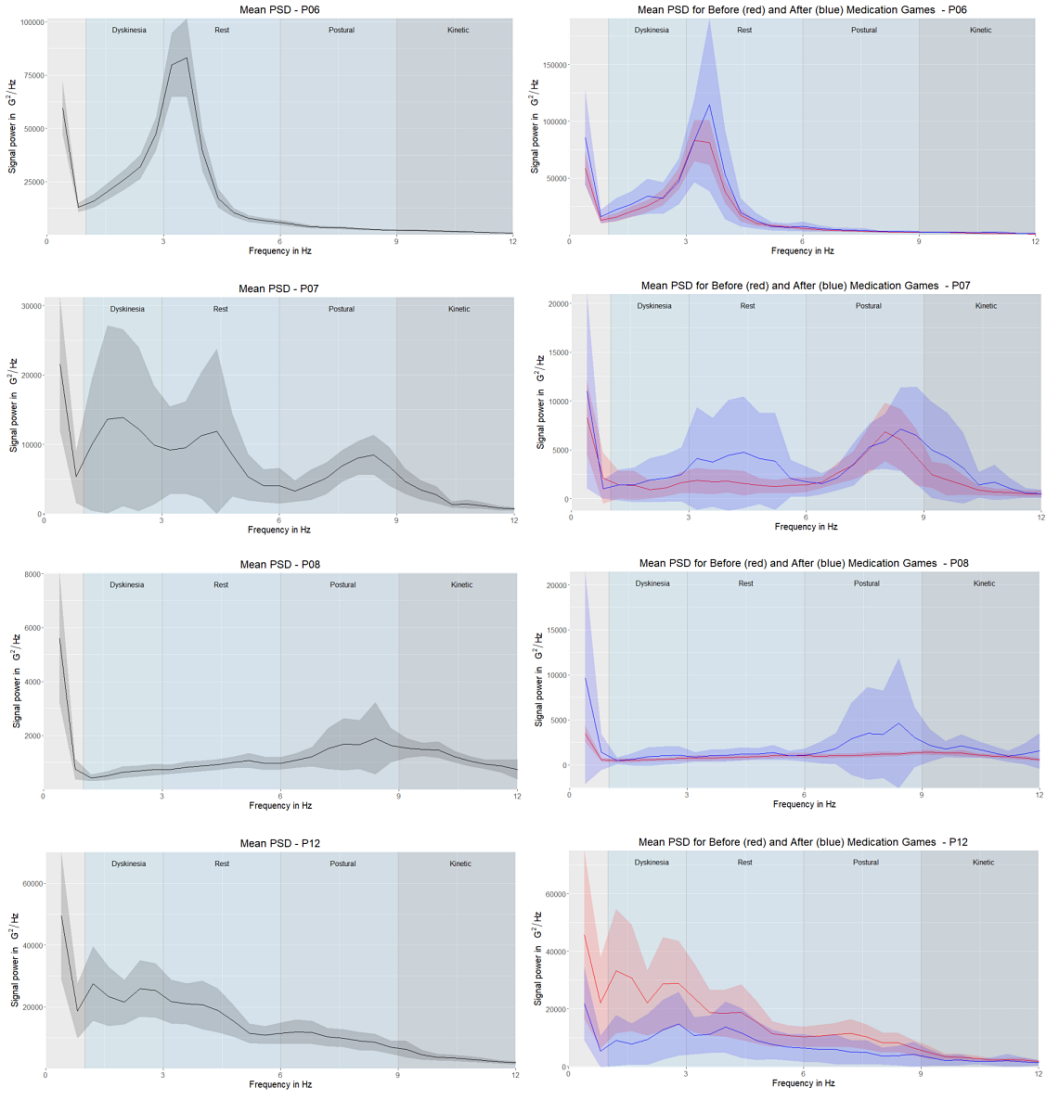
Mean of the power spectral densities with the 95% CI for the “tremor” group (P06, P07, P08, and P12). The left column shows the mean of all game sessions, and the right column shows the mean of the power spectral densities for “before” (red) and “after” (blue) games. Note that the y-axis ranges differ. Frequency areas (dyskinesia, rest tremor, postural tremor, and kinetic tremor) are denoted by different column shades of the background.

**Figure 5:**
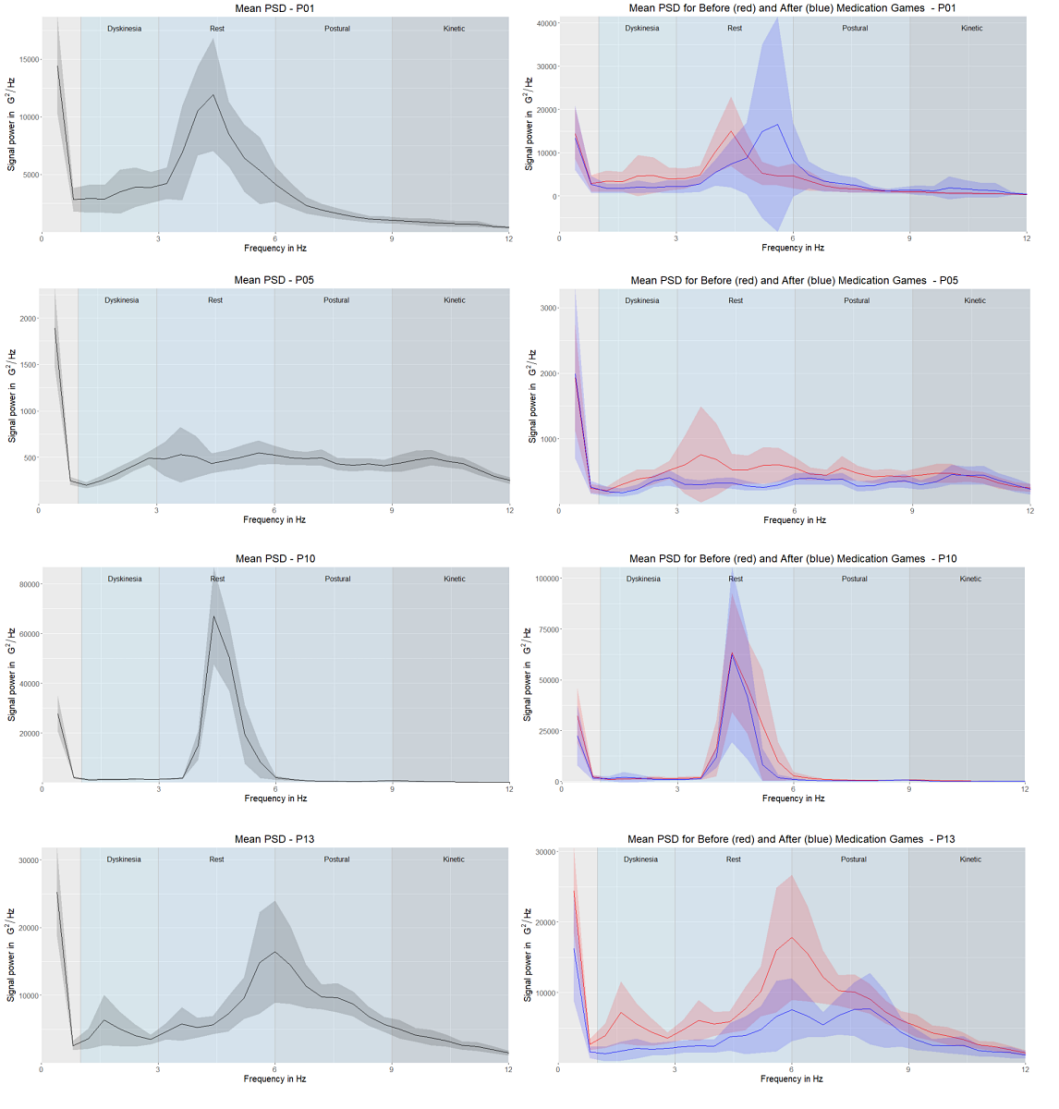
Mean of the power spectral densities (PSDs) with the 95% CI for the “hand tremor” group (P01 and P05) and the “plays with hand tremor” group (P10 and P13). The left column shows the mean of all game sessions, and the right column shows the mean of the power spectral densities for “before” (red) and “after” (blue) games. Note that the y-axis ranges differ. Frequency areas (dyskinesia, rest tremor, postural tremor, and kinetic tremor) are denoted by different column shades of the background.

We used the Welch method [27] to generate periodograms of every participant’s game sequences. This method generates a nonparametric estimation of the PSD, determining the power contained in the signal's particular frequency components (see Multimedia Appendix 2 for more details). The left columns in Figures 3-5 show the mean of all games’ periodograms as well as the confidence interval around the mean. The right columns depict the averaged periodograms for the “before” and “after” game subsets, respectively. We observed a higher PSD value in the groups with PD tremor (ie, “tremor,” “hand tremor,” and “plays with hand tremor” groups) than in the “no tremor” group. A comparison of the groups is presented in the results section.

**Figure 6:**
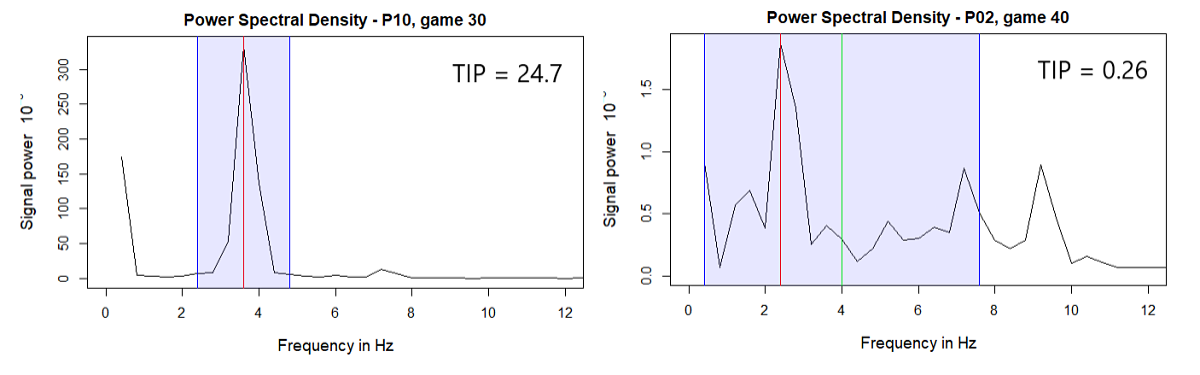
The power spectral density of one game of P10, playing with hand tremor, and one game of P02, with no tremor. The red, vertical line shows the fundamental frequency (F0), the green line shows the central frequency (F50), and the gap between the lines is the difference between F50 and F0 (|F50-F0|). For P10, F0 and F50 are the same frequency, hence, |F50-F0|=0. The blue rectangle shows the SF50 (the frequency band around F50 containing 68% of the total power of the signal). P10 has a high peak value (PV) and a narrow SF50, leading to a high tremor intensity parameter (TIP) of 24.7. The PV of P02 is small (as is the signal power in the PSD in general) and SF50 is wide; hence, he has a low TIP of 0.26. Note that the y-axis ranges in both plots differ.

From the periodograms, several features were calculated to describe the characteristics of the signal:

- area under the curve (AUC): describes the total power of the signal (Hz) [12] (Figures 3-5 present the mean of PSDs in each frequency);
- peak value (PV): represents the maximum value of the PSD;
- fundamental frequency (F0): the frequency of maximum power [5,12,16,19]. The F0 can be used to categorize the game sessions as dyskinesia, rest tremor, postural tremor, or kinetic tremor games [5,19]. The percentage of game sessions in each category of each participant is summarized in Table 2 (the red line in Figure 6 illustrates the F0);
- central frequency (F50): the central point where the periodogram is divided into two equal parts in PSD [5,12,16,19] (the green line in Figure 6 illustrates the F50);
- frequency dispersion (SF50): describes the width of the frequency band around F50 containing 68% of the total power of the signal [5,16,19] (see the blue area in Figure 6);
- |F50-F0|: the difference between F50 and F0 [5,16,19] (see the distance between the red [F0] and green [F50] lines in Figure 6);
- tremor intensity parameter (TIP): calculated as PV divided by SF50. In Figure 6, P10 has a narrow, high peak in PSD, causing a high TIP, whereas P02 has a lower PV and a wide SF50, causing a low TIP. We introduce this parameter to quantify tremor severity based on accelerometer data—a higher TIP indicates a more severe tremor.

We utilized these features to quantify tremor severity during a game session and to detect a difference in medication effects between different game sessions.

**Table 2:** The game sessions categorized as dyskinesia, rest tremor, postural tremor, or kinetic tremor according to the fundamental frequency are shown as percentages of all game sessions of each participant (the absolute number of game sessions appears in parentheses).

Participant by group		Dyskinesia	Rest tremor	Postural tremor	Kinetic tremor
**No tremor**					
	P02	52%^a^ (56/107)	7% (7/107)	41% (44/107)	0% (0/107)
	P09	18% (19/104)	62%^a^ (64/104)	20% (21/104)	0% (0/104)
	P11	64%^a^ (170/265)	34% (89/265)	2% (5/265)	0% (1/265)
**Tremor**					
	P06	30% (53/175)	70%^a^ (122/175)	0% (0/175)	0% (0/175)
	P07	10% (5/51)	10% (5/51)	80%^a^ (41/51)	0% (0/51)
	P08	30% (52/174)	12% (21/174)	58%^a^ (101/174)	0% (0/174)
	P12	34% (38/111)	40%^a^ (44/111)	26% (29/111)	0% (0/111)
**Hand tremor**					
	P01	1% (26/167)	66%^a^ (111/167)	18% (30/167)	0% (0/167)
	P05	41%^a^ (63/152)	21% (32/152)	38% (57/152)	0% (0/152)
**Plays with hand tremor**					
	P10	4% (7/169)	95%^a^ (160/169)	1% (2/169)	0% (0/169)
	P13	8% (22/282)	19% (54/282)	73%^a^ (206/282)	0% (0/282)

^a^The most prevalent symptom of the participant.

## Results

In this section, we study our two research questions using the PSD features described in the previous section: (1) how feasible is it to characterize tremor using inertial data captured during our smartphone game?, and (2) can the effects of PD medication be detected using the same inertial data captured during game sessions played before and after medication?

### Hand Tremor Characterization via the TIP: Proposal for an Objective Hand Tremor Severity Score

We found a significant correlation between self-reported UPDRS II tremor severity scores (0 to 4) and the TIP (Kendall rank correlation test: z=30.521, *P*<.001, τ=0.5367379; n=11). UPDRS II tremor scores and descriptive statistics of the TIP for each participant are shown in Table 3.

**Figure 7:**
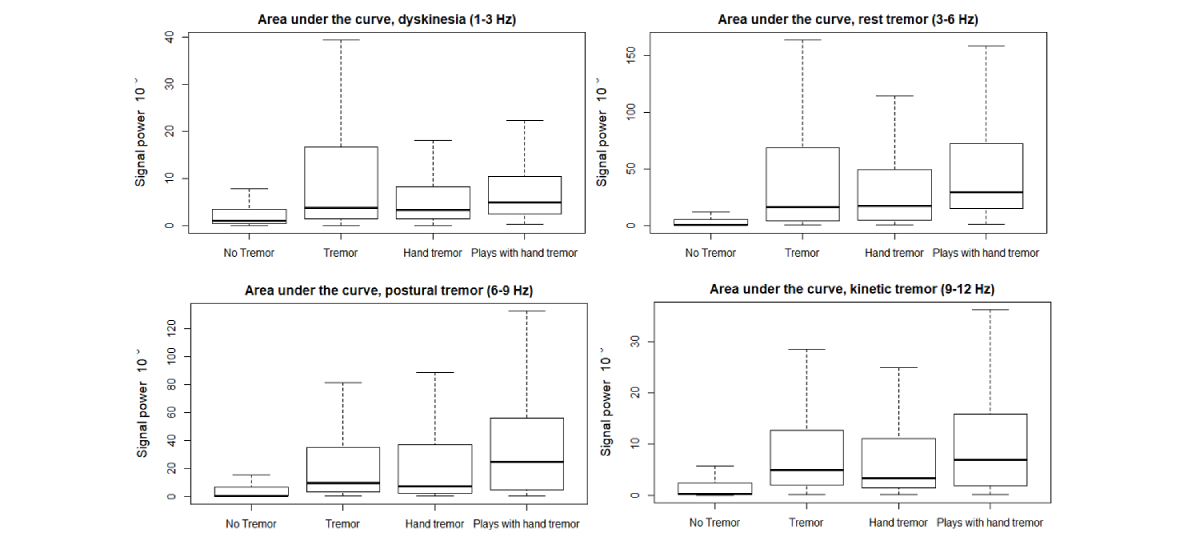
Comparison of tremor groups using area under the curve for each frequency range: dyskinesia (1-3 Hz), rest tremor (3-6 Hz), postural tremor (6-9 Hz), and kinetic tremor (9-12 Hz).

**Figure 8:**
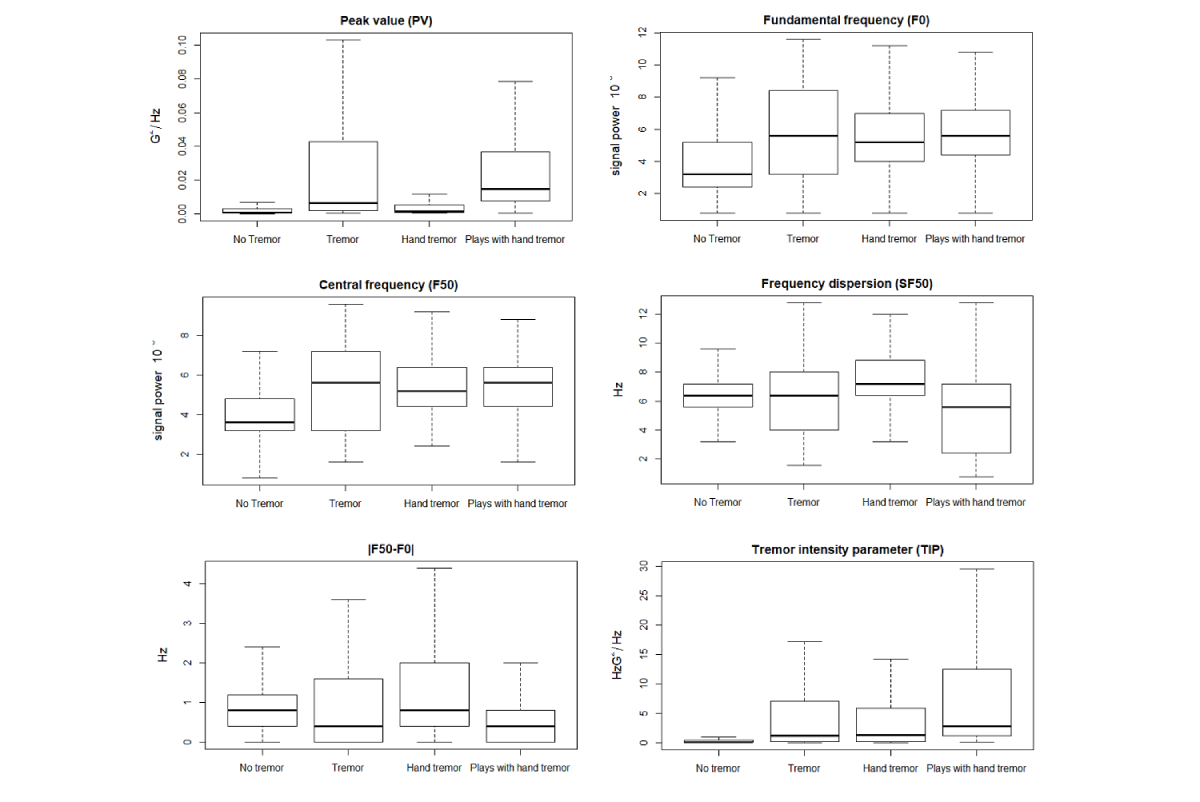
Comparison of groups in terms of the following features: peak value, fundamental frequency (F0), central frequency (F50), frequency dispersion (SF50), difference between F50 and F0 (|F50-F0|), and tremor intensity parameter.

We then compared the groups across all features (see Figures 7 and 8). Figure 7 shows the AUC for all frequency ranges. We found that for dyskinesia the means of all four groups were similar and the tremor group had the largest variability. For the other three frequency ranges (rest tremor, postural tremor, and kinetic tremor), the mean of the “plays with hand tremor” group was greater than that of the other groups. The participants in the “plays with hand tremor” group also had the highest PV and lowest SF50, and thus the highest TIP score (Figure 8). We used a Wilcoxon rank sum test to confirm that the differences between our 4 groups were statistically significant, resulting in six pairwise comparisons for AUC for all four frequency areas (dyskinesia, rest tremor, postural tremor, and kinetic tremor), PV, F0, F50, SF50, |F50-F0| and TIP (see *P* values in Table 4). Table 4 is extended in Multimedia Appendix 3, also providing the W for the Wilcoxon rank sum test.

All features were significantly different between the “no tremor” and “plays with hand tremor” groups. Additionally, all features except for SF50 showed a significant difference between the “no tremor” and “tremor” groups and between the “no tremor” and “hand tremor” groups. SF50 describes the width of the frequency band around F50 containing 68% of the total power of the signal. This suggests that when the tremor was located in a body part other than the hand holding the device, the power of the signal was spread in a wider frequency range, resembling the case with no tremor. However, the “no tremor” group differed significantly from the groups with tremors.

Features between the “tremor” and “hand tremor” groups were significantly different only in the AUC for the dyskinesia, postural tremor, and kinetic tremor frequency ranges. Hence, we can say that the effect of tremors on the accelerometer signal in these groups was mainly similar. In contrast, the comparison of the “plays with hand tremor” group with the “tremor” group and the “hand tremor“ group showed significant differences in all features except F0 and F50. The tremor effect was similar in frequency, but the magnitude of the tremors was different when the tremor hand was used for playing.

**Table 3:** Self-reported tremor severity scores using the Unified Parkinson Disease Rating Scale (UPDRS) II, item 16.

		Distribution of tremor intensity parameter					
Participant	UPDRS II item 16 score^a^	Minimum	1st quartile	Median	Mean	3rd quartile	Maximum
P02	0	0.05	0.17	0.33	0.97	0.66	22.00
P09	0	0.04	0.33	0.56	1.09	1.18	19.10
P11	0	0.01	0.03	0.04	0.05	0.06	0.31
P05	1	0.03	0.07	0.10	0.18	0.16	5.02
P07	1	0.15	0.45	1.40	5.49	2.65	81.24
P08	1	0.04	0.12	0.21	0.39	0.38	14.57
P12	1	0.07	0.71	2.05	9.47	8.81	142.83
P01	2	0.03	0.20	0.84	7.41	4.04	177.01
P10	2	0.06	3.32	15.93	59.26	59.65	769.10
P13	2	0.15	0.92	1.69	6.40	3.79	610.81
P06	3	0.31	4.97	16.54	35.48	38.12	321.28

^a^0=no tremor, 1=slight and infrequently present tremor, 2=moderate and bothersome tremor, 3=severe tremor interfering with many activities, and 4=marked tremor interfering with most activities.

**Table 4:** The *P* values from Wilcoxon rank sum tests comparing groups for each feature: area under the curve (AUC) for all four frequency areas, peak value, fundamental frequency (F0), central frequency (F50), frequency dispersion (SF50), difference between F50 and F0 (|F50-F0|), and tremor intensity parameter (TIP).

		No tremor vs tremor		No tremor vs hand tremor		No tremor vs plays with hand tremor		Tremor vs hand tremor		Tremor vs plays with hand tremor		Hand tremor vs plays with hand tremor	
AUC													
	Dyskinesia	<.001		<.001		<.001		<.001		.03		<.001	
	Rest tremor	<.001		<.001		<.001		.89		<.001		<.001	
	Postural tremor	<.001		<.001		<.001		.02		<.001		<.001	
	Kinetic tremor	<.001		<.001		<.001		<.001		.03		<.001	
Peak value			<.001		<.001		<.001		.49		<.001		<.001
F0			<.001		<.001		<.001		.62		.23		.30
F50			<.001		<.001		<.001		.43		.15		.35
SF50			.71		.54		<.001		.39		<.001		<.001
|F50 - F0|			<.001		<.001		<.001		.18		<.001		<.001
TIP			<.001		<.001		<.001		.78		<.001		<.001

### Medication Effect Detection

We investigated the effect of medication intake on the accelerometer signal characteristics. PD medication is often targeted to alleviate motor symptoms; thus, it could have affected participants’ motor performance during their game sessions. To explore this possibility, we compared the “before” and “after” game sessions of each individual.

In Figures 3-5, on the graphs on the right-hand side, we highlighted the mean PSD of “before” (red) and “after” (blue) game sessions with 95% CIs. Because our sample was relatively small, some of the 95% CI boundaries were negative [28]. In the “no tremor” group (Figure 3), P02 and P11 had peaks in the dyskinesia frequency range, and the mean PSD of “before” games was higher than the mean PSD of “after” games. This suggests that the medication partially alleviated this symptom. The 95% CIs for P09 were mostly overlapping, suggesting that there was no difference in the mean PSD of “before” and “after” games.

In the “tremor” group (Figure 4), P06 had a high peak in the rest tremor frequency area, P08 had a high peak in the postural tremor frequency area, and P07 had a peak in both. Compared with the “no tremor” group, the peaks in the “tremor” group were in tremor frequency ranges, rather than in the dyskinesia frequency range, which matches our expectation of observing this symptom. Even though the 95 CIs were overlapping, the 95% CI for “before” games was narrower. Figure 2 shows that P06 and P08 often played the game at the same time as medication intake, which might have resulted in the narrowing of the 95% CI for “before” games. P12 had two peaks in the “before” sessions in the dyskinesia frequency range; these peaks were lower in “after” sessions.

In the “hand tremor” and “plays with hand tremor” groups (Figure 5), P10 had a clear peak in the rest tremor frequency range. However, the effect of medication was not visible, since the “before” and “after” 95% CIs fully overlapped. This might indicate that the medication was working well, and its effect was maintained prior to the next intake. For P01, who suffered from hand tremor but played with his nonaffected hand, we found a difference in the tremor frequency between “before” and “after” game sessions, and the peak frequency had shifted (Figure 5). For P05 and P13, we observed that the mean PSD of “before” games was higher than of “after” games (Figure 5). P05 had a narrow 95% CI for “after” game sessions; thus, the performance was more predictable after medication intake.

In Table 5, we summarized the changes detected in all of the features between “before” and “after” game sessions. Many participants had changes in their features between the game sessions. A Wilcoxon rank sum test confirmed statistically significant differences for three participants: (1) P02 in AUC dyskinesia (W=861, *P*=.005), AUC resting tremor (W=1016, *P*<.001), AUC postural tremor (W=970, *P*=.002), AUC kinetic tremor (W=872, *P*=.036), PV (W=949, *P*=.004), SF50 (W=421, *P*=.006), and TIP (W=953, *P*=.003); (2) P09 in SF50 (W=469, *P*=.005); and (3) P11 in AUC dyskinesia (W=2767, *P*=.003), PV (W=2590, *P*=.024), F50 (W=1490, *P*=.027), SF50 (W=1327, *P*=.004), and TIP (W=2665, *P*=.011). These three participants (P02, P09, and P11) reported no tremor (UPDRS II, item 16). P02 and P09 presented with rigidity, and P11 presented with rigidity and bradykinesia; hence, it seems that the medication effect was more visible for these symptoms.

**Table 5:** Change in means of features as percentages between “before” and “after” medication game sessions. The negative values represent a lower mean in “after” game sessions than in “before” game sessions, while positive values represent the opposite.

Participant by group		AUC^a^, dyskinesia	AUC, rest tremor	AUC, postural tremor	AUC, kinetic tremor	PV^b^	F0^c^	F50^d^	SF50^e^	|F50-F0|^f^	TIP^g^
**No tremor**											
	P02	–51^h^	–51^i^	–53^h^	–56^h^	–53^h^	10	9	11^h^	24	–3^h^
	P09	52	1	–23	30	1	–13	–14	–23^h^	–28	21
	P11	–29^h^	–8	4	4	–25^h^	7	10^h^	10^h^	48	–35^h^
**Tremor**											
	P06	14	21	21	25	22	3	–4	5	31	4
	P07	45	148	8	102	4	10	2	1	2	–8
	P08	41	28	142	45	139	1	0	3	4	108
	P12	–62	–40	–48	–30	–59	–14	–9	0	–4	–69
**Hand tremor**											
	P01	–52	9	51	104	2	7	9	–5	–24	–3
	P05	–26	–52	–26	–7	–45	13	–3	1	23	–51
**Plays with hand tremor**											
	P10	1	–23	–52	–42	–21	–1	–2	7	61	–3
	P13	–63	–53	–41	–34	–51	–3	1	1	–27	–65

^a^AUC: area under the curve.

^b^PV: peak value.

^c^F0: fundamental frequency.

^d^F50: central frequency.

^e^SF50: frequency dispersion.

^f^|F50-F0|: difference between F50 and F0.

^g^TIP: tremor intensity parameter.

^h^Difference is statistically significant at *P*<.05, based on the Wilcoxon rank sum test.

^i^Difference is statistically significant at *P*<.001, based on the Wilcoxon rank sum test.

## Discussion

In this paper, we show that it is feasible to detect and characterize PD hand tremor severity using accelerometer data captured during game play. Further, we investigated the medication effect on the accelerometer signal, demonstrating a statistically significant difference in the accelerometer data characteristics of the game sessions played before and after medication intake by participants with rigidity and bradykinesia.

### Revisiting the Research Questions

First, how feasible is it to characterize hand tremor using inertial data captured during our smartphone game? To this end, we introduced the TIP for characterizing hand tremor severity, as computed using accelerometer data. We show that TIP is significantly correlated with the tremor score (item 16) on the UPDRS II [9]. TIP was significantly different between participants with no tremor and those with tremor symptoms, as well as between the participants playing with the tremor hand and participants with tremor in the opposite hand or in other body parts (Table 4). These results suggest that it is possible to objectively detect and quantify the severity of hand tremor using smartphone accelerometer data across different tremor types and intensities.

Inspired by previous work in hand tremor analysis using accelerometer data [5,11-16,18,19], we analyzed the accelerometer data collected during a 1-month real-world trial of the STOP app [21]. Earlier studies have already shown that accelerometer signals can be used to measure tremor under controlled conditions similar to traditional clinical assessments using the UPDRS II, either by discriminating between people with and without PD [11-13,15,18] or by measuring tremor severity [5,14,16,19]. We used partially similar methods to those used in previous studies [5,12,16,19], but in contrast, we focused on the feasibility of objective assessments in daily life, with a task that could be conducted anywhere in less than 30 seconds using one’s own smartphone. The smartphone is always with you, and a gamified task does not draw attention, even in public places. The low burden enables regular monitoring, providing continuous data of symptoms over time to support in treatment decisions.

Second, can the effects of PD medication be detected using the same inertial data captured during game sessions played before and after medication intake? In other words, we explored whether or not medication-induced changes in motor symptoms could be measured using frequency-domain features extracted from accelerometer data. We classified the games played into two groups: “before” and “after” medication intake. For participants suffering from rigidity and bradykinesia, we found a statistically significant difference in particular signal characterizing features (Table 5). It is known that bradykinesia is usually responsive to PD medication [29]. Kostikis et al [14] also compared “off” and “on” medication states in laboratory conditions with two volunteers with PD. Even though they did not measure rigidity, according to the physician observing the measurements, the patients’ rigidity improved after medication intake. Hence, our results are in line with their observations. Further research is needed to reproduce these findings and to investigate why we could not find a before-and-after difference for all participants and for tremor symptoms. To this end, we hypothesize that the time window of measurement could have had an effect on the results. It is possible that our participants were consistently under the effects of their medication, thus resulting in similar data across all game sessions.

### Reflection on Smartphone-Based Monitoring of PD in Real Life

As this study was not a laboratory-controlled experiment, the way participants played the game could have affected the accelerometer data signal. For example, if the participant’s hand was extended, such a position might have induced postural tremor, or if the arm was resting on their lap, rest tremor might have become dominant. Table 2 categorized the game sessions according to the F0 as dyskinesia, rest tremor, postural tremor, or kinetic tremor, as determined in a study by Pierleoni et al [5]. Indeed, based on the interview data reported in our previous study [21], some participants implemented strategies to “beat the game.” For example, P10 and P12 reported to press their elbow against their torso to keep their hands steady, and P01 would occasionally play the game while holding the smartphone with both hands. These three participants had the most games classified as rest tremor (Table 2). P08 mentioned that the game was “easier” if sitting down, and a majority of his games were grouped as postural tremor. P07 noticed how his posture impacted the game and held his hand in such a way that it wasn't supported by his body; very likely as a consequence, 80% of his games were within the F0 postural tremor frequency area.

It should be noted that the F0 in tremor frequencies does not indicate tremor (Table 2). F0 indicates the frequency of maximum power in PSD but does not otherwise take into account the magnitude of the peak. The TIP describes the severity of the tremor effect, and with the F0 we can further characterize the type of tremor.

### Limitations

With the STOP app, tremor analysis is limited to tremor severity in participants’ hands, measured using their own smartphone as the instrument. Conversely, in assessments by health professionals, tools such as the UPDRS can be used to evaluate other tremor characteristics, such as amplitude in the legs, jaw, and neck. In our study, the fragmentation of the smartphone device base already caused minor issues, and this can only be expected to exacerbate in the future. To this end, measures to also track and account for the exact device make and model should be added to the approach.

Levodopa treatment is prescribed to alleviate motor symptoms, and although we know the medication intake time, we often ignore the symptoms in particular participants that the medication was prescribed for; hence, the analysis of the medication intake effect is preliminary (it is unclear whether or not the medication was meant to reduce tremor severity). Additionally, the time difference between game sessions and medication intake (“before” and “after” game sessions) varied, as did the magnitude of the changes induced by the medication, which were recorded by the STOP app. This time difference should be taken into account in future studies.

The participant sample size was admittedly small. However, this was compensated for by the high number of individual contributions in the form of game sessions. Further, our data analysis focused on results that generalize sufficiently well for the purposes of this paper: investigating the role of accelerometer data in differentiating between different symptoms and the effects of medication.

### Future Work

Further research is needed to assess the internal and external validity of the TIP, as our results suggest it has the potential to quantify tremor severity. Previous studies [5,14,16,19] have based their tremor severity evaluations on the UPDRS, which evaluates the tremor on a scale from 0 to 4. Similarly, we used the self-assessed UPDRS II as a baseline in our measurements. However, the UPDRS was designed not for daily symptom severity assessment but rather to detect changes in symptom level in the long term. In future, we shall explore different alternatives to quantify tremor that we can use for baseline. One option is to utilize self-reporting about tremor severity, or to compare the TIP of a game session to the user’s long-term average to provide insight into the variation in personal tremor severity levels.

Changes in our accelerometer features were inconsistent between “before” and “after” medication game sessions. This suggests that we could explore personalized tremor classification models. The effect of hand tremor is visible in the accelerometer signal, but we did not find a statistically significant effect of medication. In addition, it is necessary to focus on particular medication types and PD symptoms to explore the difference between “before” and “after” medication game sessions using accelerometer data in more homogeneous conditions.

Given the availability and sensing capabilities of smartphones, we envision that tools such as the STOP app can support the care and monitoring of PD as well as enable frequent, or even continuous, measuring of medication effects in naturalistic conditions. Even though real-life assessments pose a challenge for data quality due to differences in sensing devices and conditions, standalone smartphone solutions can have a lower burden, thus increasing engagement. For clinicians, a richer picture of symptom severity enabled by sensor data could enable them to better understand people’s conditions and prescribe tailored medications.

### Conclusions

In summary, it is feasible to detect and quantify the severity of hand tremor using accelerometer data collected with modern, off-the-shelf smartphones. We replicated and validated previously reported features derived from accelerometer data collected in real-world settings. To this end, we presented the TIP, a metric that could support further research into unobtrusive tremor assessment with smartphones but requires further internal and external validation. Additionally, we identified a statistically significant difference between the game sessions before and after medication intake among participants with rigidity and bradykinesia, and concluded that detecting the effects of PD medication is possible but further research is warranted.

## Appendix

Multimedia Appendix 1 Participants' details.

Multimedia Appendix 2 Technical details of the linear interpolation of the accelerometer signal and the Welch method.

Multimedia Appendix 3 Wilcoxon rank sum test details of group comparisons.

## Abbreviations

AUC: area under the curve
F0: fundamental frequency
F50: central frequency
|F50-F0|: difference between F50 and F0
PD: Parkinson disease
PSD: power spectral density
PV: peak value
SF50: frequency dispersion
STOP: Sentient Tracking of Parkinson
TIP: tremor intensity parameter
UPDRS: Unified Parkinson Disease Rating Scale
