# Supplementary material for: Smartphone-Based Monitoring of Parkinson Disease: Quasi-Experimental Study to Quantify Hand Tremor Severity and Medication Effectiveness
Source: JMIR Mhealth Uhealth. 2020 Nov 26;8(11):e21543. doi: 10.2196/21543 (PMC7728543; doi:10.2196/21543)
Supplement: Multimedia Appendix 1 [file mhealth_v8i11e21543_app1.pdf]

Multimedia Appendix 1

Participant details: Age, how many years they have had the PD diagnosis, the number of PD medication, and the number of daily medication intake times, self-assessed UPDRS II (Unified Parkinson's Disease Rating Scale) scores (general and tremor, item 16), do they have a deep brain stimulator (DBS) installed, do they suffer from hand tremor, and do they play with the tremor hand, and on last row, other symptoms affecting to the hand's motor performance. The UPDRS II score describes the level of PD symptoms affecting daily activities (max. 52). Scale for tremor item in UPDRS: 0 - no tremor, 1 - slight and infrequently present tremor, 2 - moderate and bothersome tremor, 3 - severe tremor interfering many activities, 4 - marked tremor interfering with most activities.

	**P01**	**P02**	**P05**	**P06**	**P07**	**P08**	**P09**	**P10**	**P11**	**P12**	**P13**
**Age**	69	52	67	65	58	71	72	66	65	73	54
**Years since PD diagnosed**	6	3	2	17	6	5	10	5	3	13	8
**Number of PD medication**	2	4	1	5	4	1	4	3	2	4	3
**Number of total daily medication intakes**	1	5	2	7	5	4	5	4	4	5	5
**UPDRS II score**	13	5	13	31	11	9	16	10	3	8	11
**Tremor on UPDRS**	2	0	1	3	1	1	0	2	0	1	2
**Deep Brain Stimulator (DBS) installed**	No	No	No	Yes	No	No	No	No	No	No	Yes
**Suffer from hand tremor**	Yes	No	Yes	No	No	No	No	Yes	No	No	Yes
**Plays with tremor-affected hand**	No	-	No	-	-	-	-	Yes	-	-	Yes
**Other issues affecting hands**	-	Rigidity in hands	Right-handed, plays with left	-	-	-	Rigidity in wrists	-	Rigidity, bradykinesia	-	-
