# Supplementary material for: Smartphone-Based Monitoring of Parkinson Disease: Quasi-Experimental Study to Quantify Hand Tremor Severity and Medication Effectiveness
Source: JMIR Mhealth Uhealth. 2020 Nov 26;8(11):e21543. doi: 10.2196/21543 (PMC7728543; doi:10.2196/21543)
Supplement: Multimedia Appendix 2 [file mhealth_v8i11e21543_app2.pdf]

Multimedia Appendix 2

The Linear Interpolation of the Accelerometer Signal

Accelerometer data was recorded as the participants played a game, the sampling rate was programmatically requested to be 50 Hz, to cover the PD tremor frequency range and to prevent aliasing based on the Nyquist-Shannon theorem [1]. To address the sensors' sampling variation and get uniformly sampled data, we applied a linear interpolation on the accelerometer signal. We calculated the Euclidean Norm $\sqrt{x^2 + y^2 + z^2}$ of the three-dimensional (x, y, z) acceleration signal, de-trended the signal by removing the DC offset, and filtered the time series with a 5th order Butterworth bandpass filter and cut-off frequencies at 0.5 Hz and 15 Hz.

The Welch Method

We analyzed the accelerometer data in the frequency domain using the Welch method [2] to generate periodograms of every participant's game sequences. This method generates a non-parametric estimation of the Power Spectral Density (PSD) and, in contrast to the direct application of a discrete Fourier transform, it reduces the variance with increasing number of samples by averaging multiple sub-spectra of overlapping window intervals. In this way, the Welch method highlights continuously present frequency components while stochastic, irregularly occurring ones, such as noise interference, are suppressed.

In practice, each 10-second sequence is split up into 8 2.222 second overlapping segments. After a detrending of the segments, the Hamming window function is applied to minimize the sidelobes caused by the window's frequency response. Each segment's frequency spectrum is derived using the Fast Fourier Transform (FFT) algorithm and exhibits a frequency resolution of about 0.4Hz. Finally, those sub-spectra are fused to a single periodogram through a bin-wise averaging.

1. Shannon, C.E., *Communication in the presence of noise.* Proceedings of the IRE, 1949. **37**(1): p. 10-21.
2. Welch, P., *The use of fast Fourier transform for the estimation of power spectra: a method based on time averaging over short, modified periodograms.* IEEE Transactions on audio and electroacoustics, 1967. **15**(2): p. 70-73.
