# Supplementary material for: Smartphone-Based Monitoring of Parkinson Disease: Quasi-Experimental Study to Quantify Hand Tremor Severity and Medication Effectiveness
Source: JMIR Mhealth Uhealth. 2020 Nov 26;8(11):e21543. doi: 10.2196/21543 (PMC7728543; doi:10.2196/21543)
Supplement: Multimedia Appendix 3 [file mhealth_v8i11e21543_app3.pdf]

Multimedia Appendix 3

Wilcoxon rank sum test details of group comparisons: The *P* and W values from Wilcoxon rank sum test when comparing groups "No tremor" (denoted as NoTr), "Tremor" (Tr), "Hand tremor" (HTr), and "Plays with hand tremor" (PHTr) for each feature; AUC for all four frequency areas (dyskinesia, rest, postural, kinetic), PV, F0, F50, SF50, |F50-F0| and TIP.

	NoTr vs Tr	NoTr vs HTr	NoTr vs PHTr	Tr vs HTr	Tr vs PHTr	HTr vs PHTr
AUC, dysk., *P* (W)	<.001 (95554)	<.001 (279269)	<.001 (176311)	<.001 (480937.5)	.03 (326592.5)	<.001 (209757.5)
AUC, rest, *P* (W)	<.001 (58585)	<.001 (327848)	<.001 (209449)	.89 (525131.5)	<.001 (382726.5)	<.001 (224180.5)
AUC, post., *P* (W)	<.001 (28556)	<.001 (299059)	<.001 (190076)	.02 (494993.5)	<.001 (358996.5)	<.001 (213804.5)
AUC, kin., *P* (W)	<.001 (50412)	<.001 (308516)	<.001 (186968)	<.001 (466401.5)	.03 (326672.5)	<.001 (206010.5)
PV, *P* (W)	<.001 (53712)	<.001 (321968)	<.001 (210707)	.49 (517502.5)	<.001 (390830.5)	<.001 (229568.5)
F0, *P* (W)	<.001 (68626.5)	<.001 (279591)	<.001 (166081)	.62 (533725)	.23 (317791.5)	.3 (184325.5)
F50, *P* (W)	<.001 (67504)	<.001 (291090)	<.001 (171760)	.43 (537788)	.15 (319887)	.35 (83760)
SF50, *P* (W)	.71 (28077)	.54 (189115.5)	<.001 (85810)	.39 (514983.5)	<.001 (243630)	<.001 (145803)
|F50 - F0|, *P* (W)	<.001 (90239.5)	<.001 (161919.5)	<.001 (76225.5)	.18 (509067.5)	<.001 (251479)	<.001 (152991)
TIP, *P* (W)	<.001 (2309)	<.001 (315302)	<.001 (209433)	.78 (523156.5)	<.001 (392883.5)	<.001 (228775.5)
